# FLAIRectomy: Resecting beyond the Contrast Margin for Glioblastoma

**DOI:** 10.3390/brainsci12050544

**Published:** 2022-04-25

**Authors:** Alexander F. Haddad, Jacob S. Young, Ramin A. Morshed, Mitchel S. Berger

**Affiliations:** Department of Neurological Surgery, University of California San Francisco, 505 Parnassus Ave, M-779, San Francisco, CA 94143, USA; alexander.haddad@ucsf.edu (A.F.H.); jacob.young@ucsf.edu (J.S.Y.); ramin.morshed@ucsf.edu (R.A.M.)

**Keywords:** glioblastoma, resection, extent of resection, flair

## Abstract

The standard of care for isocitrate dehydrogenase (IDH)-wildtype glioblastoma (GBM) is maximal resection followed by chemotherapy and radiation. Studies investigating the resection of GBM have primarily focused on the contrast enhancing portion of the tumor on magnetic resonance imaging. Histopathological studies, however, have demonstrated tumor infiltration within peri-tumoral fluid-attenuated inversion recovery (FLAIR) abnormalities, which is often not resected. The histopathology of FLAIR and local recurrence patterns of GBM have prompted interest in the resection of peri-tumoral FLAIR, or FLAIRectomy. To this point, recent studies have suggested a significant survival benefit associated with safe peri-tumoral FLAIR resection. In this review, we discuss the evidence surrounding the composition of peri-tumoral FLAIR, outcomes associated with FLAIRectomy, future directions of the field, and potential implications for patients.

## 1. Introduction

**Figure 1:**
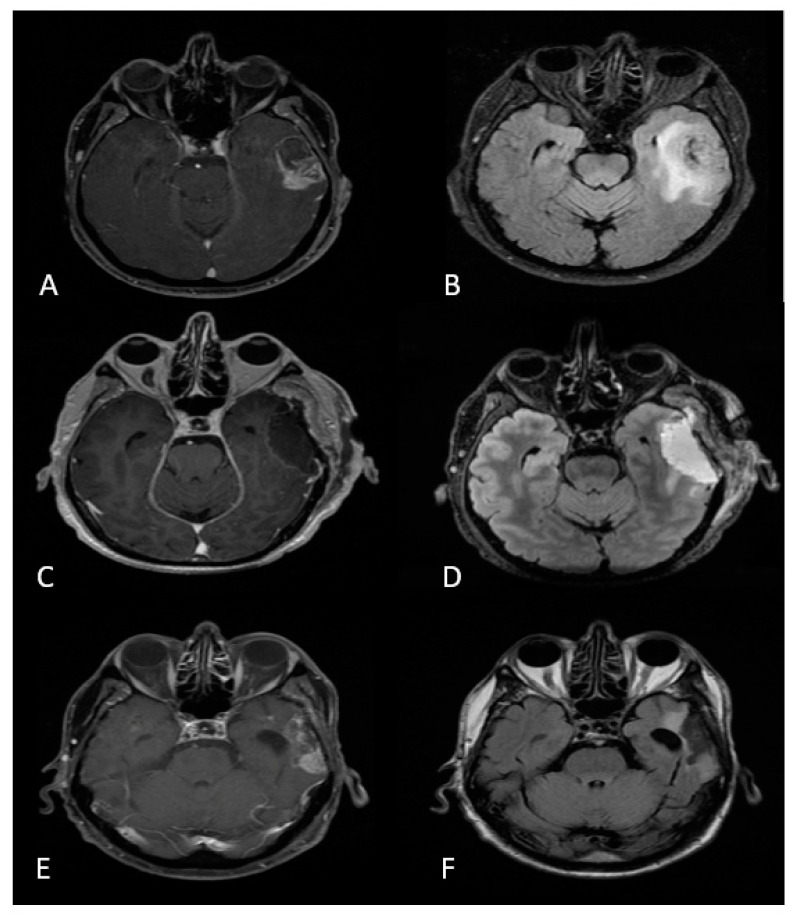
71-year-old man with left temporal glioblastoma. (**A**,**B**) Preoperative T1 post-contrast and T2 FLAIR MRI demonstrating large ring enhancing left temporal lesion. (**C**,**D**) Postoperative T1 post-contrast and T2 FLAIR MRI highlighting gross total resection of the contrast enhancing lesion, with some minimal residual FLAIR signal. (**E**,**F**) 11-month postoperative T1 post-contrast and T2 FLAIR MRI showing a contrast enhancing lesion in the left temporal lobe near the resection cavity consistent with recurrence.

IDH (isocitrate dehydrogenase)-wildtype glioblastoma (GBM) is the most common primary malignant brain tumor and is highly aggressive, with a dismal prognosis despite standard of care treatment including surgical resection, radiation, and chemotherapy [1,2]. Surgical resection for GBM has primarily focused on maximal resection of the contrast-enhancing tumor and subsequent survival benefits [3]. Yet despite gross total resection of the contrast enhancing tumor, most patients experience recurrence near the resection bed (Figure 1). This has sparked recent investigation into whether resecting the non-contrast enhancing FLAIR positive tumor, or FLAIRectomy, is of benefit. In this article, we discuss the composition of peri-tumoral FLAIR in the context of GBM, studies investigating the role of FLAIR resection, and the future of FLAIRectomy. 

## 2. What Is FLAIR?

Magnetic resonance imaging (MRI) is a crucial tool utilized by neurosurgeons and neuro-oncologists to assess GBM tumors, including in the preoperative, postoperative, and even intraoperative settings [4,5]. Contrast enhanced T1- weighted imaging can aid in providing insight into the presence of blood brain barrier breakdown, with contrast enhancement potentially suggesting a higher grade lesion. However, T2- weighted sequences are much more sensitive to differences the water content of the brain [4]. Fluid-attenuated inversion recovery (FLAIR) T2 weighted images involve the suppression of T2 signal from cerebrospinal fluid (CSF) in order to better highlight pathologic processes. FLAIR changes on imaging have been suggested to have an increased sensitivity in detecting leptomeningeal spread of tumors and non-contrast enhancing lesions [4]. FLAIR hyperintensity on imaging often extends beyond the contrast-enhancing portion of a GBM tumor, and there is growing interest into the tumor microenvironment composition within areas of FLAIR change [6,7,8].

There is a relative paucity of studies comparing regional microscopic histopathological tumor invasion with preoperative MRI features. A 2010 study by Yamahara et al. evaluated seven autopsy brains of GBM patients, finding that significant tumor cell infiltration was detected up to 14 mm from the tumor border as defined by the contrast enhancing area on MRI [9]. This finding was further supported in a study of 119 GBM tissue specimens by Barajas et al. who found that >80% of non-enhancing samples contained evidence of tumor cells on histopathologic analysis [10]. Most studies have demonstrated higher cellularity within areas of enhancing regions compared to non-enhancing regions [10,11]. In contrast, a recent study of 37 patients with GBM by Eidel et al. using stereotactic biopsy specimens of tumor tissue found that non-enhancing tissue had the highest content of viable tumor cells with a similar average cell density to contrast enhancing tissue [12].

One limitation of these prior studies is that a comprehensive microenvironment evaluation of the FLAIR region was not performed. Focus has been given to the non-enhancing tumor area just beyond the enhancing edge of the tumor. Yet, FLAIR signal also represents edema and inflammatory milieu, and it is still unclear to what degree tumor invasion versus inflammation account for these imaging changes. Wurtemberger et al. compared the histopathologic features of T2 hyperintense regions on MRI between GBM and metastasis, specifically evaluating edema pattern and tumor infiltration; they found that perilesional T2 hyperintensities in GBM contained additional tumor infiltration while perimetastatic T2 hyperintensities were more likely composed of increased free water and vasogenic edema [13]. The FLAIR characteristics of tumor infiltration and edema also differ significantly. Edema is more commonly found in the white matter and respects the cortical ribbon while FLAIR associated with gliomas and tumor infiltration frequently involves gray matter and can cause some parenchymal expansion [14,15,16]. In addition, FLAIR signal associated with edema can be more hyperintense than when it is associated with glioma tumor infiltration [17].

Unsurprisingly perhaps, in light of the aforementioned studies, the extent of non-contrast enhancing tumor associated with a GBM has also been correlated with patient survival in specific circumstances. Jain et al. found that the presence of non-enhancing GBM signal crossing the midline was associated with worse survival [18]. Similarly, in a study of 151 GBM patients, Lasocki et al. demonstrated that increased non-enhancing cortical signal abnormality was associated with worse survival, albeit in peripherally located lesions [19].

In summary, FLAIR signal in GBM contains infiltrative tumor cells and the amount of FLAIR signal abnormality surrounding a GBM is correlated with survival in specific circumstances. In addition, the peri-resection cavity location of most recurrences suggests that the infiltrative tumor cells present in FLAIR play a key role in the progression and recurrence of GBM, even those with gross total resection of the contrast-enhancing lesion. These findings support additional consideration surrounding the resection of GBM associated FLAIR signal and even normal appearing tissue.

## 3. FLAIRectomy

Most studies in GBM thus far have focused on the resection of contrast-enhancing tumor with regards to patient survival and outcomes. Early evidence of the benefit of aggressive surgical resection for glioblastoma was established in a landmark paper from Lacroix et al. in 2001 demonstrating that removal of over 98% of the enhancing tumor was an independent predictor of longer overall survival [3]. Although this data was interpreted by some surgeons to indicate an “all-or-nothing” strategy was appropriate when considering resection of lesions concerning for glioblastoma, the benefit of surgical resection appeared to demonstrate a “dose-dependent” effect beginning at 89% resection within the entire cohort. Sanai et al. built upon these findings by demonstrating that a stepwise increase in survival was observed for patients with newly diagnosed GBM with increasing volumetric extent of resection [20]. This observed benefit held true when greater than 78% of the enhancing tissue was resected. These prior retrospective studies are further supported by prospective data from the recent fluorescence-guided surgery trials using 5-aminolevulinic acid (5-ALA) [21]. The use of 5-ALA increased the extent of resection from 36% to 65% in the trial, and accordingly there was an improvement in the 6-month progression free survival (41% vs. 21%) and overall survival (16.7 months vs. 11.8 months), again highlighting the benefit of maximal resection of contrast-enhancing tissue.

While these studies provided strong evidence for the benefit of removal of the contrast enhancing tumor, they neglect to evaluate the removal of non-contrast enhancing tumor on patient outcomes. One key aspect of supratotal resections is they aim to reduce the residual tumor burden for the enhancing and non-enhancing tumor, which is critical as the smallest residual tumor volumes for both have been found to be a key predictor of the best patient outcomes. In a retrospective review of 128 GBM patients, Grabowski et al. found that contrast enhancing residual tumor volume, T2 FLAIR residual tumor volume, and overall extent of resection were significant predictors of survival when controlling for age and KPS [22]. The impact of residual non-enhancing tumor on overall survival was further supported in a subsequent multicenter study of 134 GBM patients by Kotrotsou et al.; they found that patients with low residual non-enhancing tumor volume after surgical resection (<70.2 cm^3^) had a significant survival benefit of 5.6 months [23]. The amount of FLAIR abnormality resection for optimizing outcomes for patients with GBM varies between groups. In a study of 1229 patients, the MD Anderson team reported resection of over 53% of the FLAIR abnormality, along with complete resection of the contrast enhancing tumor, as being associated with the best outcomes [24]. Others groups have reported that resection of 20–45% of the FLAIR signal beyond the contrast-enhancing tumor was associated with improved overall survival, suggesting the threshold of resection of FLAIR tumor necessary to improve survival may not be as high as previously thought [25,26].

Many of these studies do not consider the underlying molecular subgroups of patients with GBM. Molinaro et al. examined the impact of supratotal resections that extend into the non-enhancing, FLAIR abnormality for 761 patients with GBM in the molecular era [27]. In this cohort, patients younger than 65 years of age with newly diagnosed IDH-wildtype GBM who had resections resulting in less than 5.4 mL of residual non-enhancing FLAIR disease had outcomes that were the same as patients diagnosed with IDH-mutant glioblastoma, an entity that would no longer be considered GBM in the latest World Health Organization classification schema [28]. Interestingly, Molinaro et al. also found that older patients with IDH-wild type tumors primarily benefit from aggressive reduction of contrast enhancing tumor. These findings highlight the necessity of integrating molecular data into decisions surrounding tumor resection. While this data can be difficult to obtain preoperatively, novel techniques are currently being evaluated that might provide neurosurgeon oncologists with information on underlying tumor molecular characteristics pre- or intraoperatively [29,30,31,32].

Finally, some groups have even reported performing lobectomies when feasible in non-eloquent cortex such as the right frontal or temporal lobe to ensure removal of all the enhancing and non-enhancing tumor, as well as some normal appearing brain on MRI scan, and found significantly improved progression free (11.5 vs. 30.7 months) and overall survival (18.7 and 44.1 months) when compared to traditional gross total resection of the contrast enhancing tumor [33].

The benefits of maximal resection have to be weighed against the risks of new postoperative neurological deficit, which can also have a significant impact on overall survival. A number of studies have demonstrated reduced overall survival in patients with GBM who suffered from a new neurologic deficit in the postoperative setting [34,35]. Indeed, a study of 115 GBM patients by Rahman et al. demonstrated that the survival benefit of 95% extent of resection was offset by a new postoperative neurological deficit [34]. However, previous studies have been limited by a lack of molecular tumor characteristics and information surrounding non-contrast enhancing tumor resection. Aabedi et al. sought to further investigate the relationship between new postoperative neurological deficit and overall survival in a homogenous group of 228 patients with IDH wild-type GBM with detailed clinical and surgical data, including extent of non-contrast enhancing tumor resection [36]. Similarly to Rahman et al., they found that a new postoperative neurological deficit was a key mediator of overall survival, especially motor deficits; elderly patients over the age of 60 were also the most susceptible to the negative prognostic impact of a new postoperative deficit with a median survival of 11.6 months with one or more deficits regardless of extent of resection [36]. These findings further support the role of maximal safe resection when possible and the limitation of new postoperative deficits through the use of technologies such as intraoperative navigation and mapping techniques [37]. In addition, overall patient functional outcome, including but not limited to postoperative neurological deficit, should be carefully considered, with a need for improved standardized methods to measure and report postoperative clinician and patient reported outcomes [38].

## 4. Future Directions

**Figure 2:**
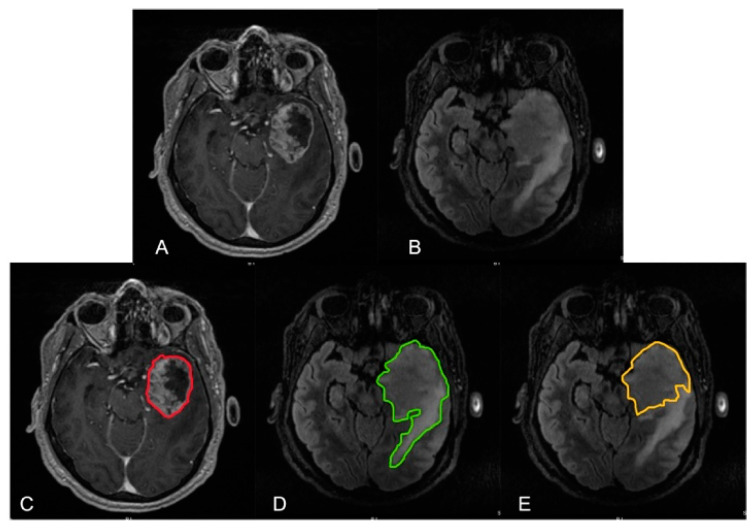
Preoperative magnetic resonance imaging highlighting left temporal lobe glioblastoma. (**A**) T1 post-contrast MRI. (**B**) T2 FLAIR MRI. (**C**) Red line highlighting contrast-enhancing tumor. (**D**) Green line demonstrating extent of tumor associated FLAIR. (**E**) Gold line outlining potential extent of non-contrast enhancing tumor or mass-like FLAIR signal.

A limitation of studies evaluating the benefit of non-contrast enhancing tumor or T2 FLAIR tumor resection is inconsistent definitions of these volumes, potentially contributing to variability in outcomes in the literature (Figure 2). It can also be difficult to distinguish between tumor associated edema and non-contrast enhancing tumor on conventional MRI; there is certainly overlap between the two entities. Advanced imaging techniques may potentially be able to provide more detailed information regarding the extension of non-contrast enhancing tumor. MR perfusion imaging may aid in the identification of non-contrast enhancing tumor by assessing relative cerebral blood volume (CBV) elevations outside of the contrast enhancing tumor. CBV has been correlated with tumor vascularity and cell density [39,40]. In addition, the combination of elevated CBV and restricted diffusion of tissue has been shown to correlate with the eventual development of contrast-enhancement [41]. There has also been some thought surrounding the use of MR spectroscopy to evaluate the presence of non-contrast enhancing tumor (increased choline corresponding to increased cell membrane turnover and reduced N-acetylaspartate). However, the utility of MR spectroscopy in the postoperative setting to quantify extent of resection is unclear. Positron emission tomography–computed tomography (PET CT) with amino acid tracers such as [^18^F]-fluoroethyl-L-tyrosine (FET) or L-( methyl-[^11^C]) methionine (C-MET) have also shown some promise in identifying non-contrast enhancing tumor, albeit in a limited number of studies [42,43]. Finally, machine learning techniques have also been evaluated as potential aids in the determination of non-contrast enhancing tumor [19,44,45]. Artzi et al. demonstrated the ability of a support-vector-machine algorithm to correctly identify infiltrative tumor vs. vasogenic edema (based on senior neuroradiologist read) with an accuracy of 87%, highlighting the potential promise of these techniques, but also the need for histologic validation of imaging findings [45].

Technologies also exist for the identification of non-contrast enhancing tumor tissue intraoperatively. This includes Raman-based intraoperative imaging methods which provide detailed information regarding tumor infiltration and pathology in the operating room [46,47,48,49]. Raman-based imaging methods may also potentially allow for the rapid intraoperative determination of ideal resection margins, with a recent study showing the ability of Stimulated Raman scattering (SRS) to provide a brain tumor diagnosis in under 150 s [50]. Intraoperative flow cytometry and desorption electrospray ionization (DESI) have also shown some preliminary promise in the intraoperative identification of tumor cells and may potentially contribute to the identification of infiltrating tumor cells and tumor margins [51,52,53,54,55]. These tools may aid in the maximal safe resection of GBM in the future.

While primarily utilized to identify contrast enhancing tumor, 5-ALA may also be of use in the intraoperative identification of infiltrating tumor cells beyond the contrast enhancing tumor edge [56,57]. Studies have shown that 5-ALA fluorescence can include non-contrast enhancing tumor and, unsurprisingly, that tumor resections guided by 5-ALA go past contrast enhancing tumor identified on preoperative MRI [58,59]. Indeed, in a study of 34 patients with high grade glioma by Coburger et al. 5-ALA was found to have a higher sensitivity and specificity for detecting infiltrating tumor at the edge of a resection cavity than intraoperative MRI [60]. However, the negative predictive value of 5-ALA fluorescence varies significantly in the literature and likely decreases with an increasing distance from the majority of the tumor, limiting the full identification of non-contrast enhancing tumor [56]. To potentially partially counteract this, the sensitivity of 5-ALA for infiltrating tumor may be enhanced by the concurrent use of spectroscopy or confocal microscopy [61,62,63]. Novel tumor specific fluorescent tags, such as labeled antibodies against EGFR, [64,65] labeled chlorotoxin, [66] and labeled proteoglycan glypican-1 (GPC-1) antibodies [67] may contribute to the identification of infiltrating tumor in the future, but still require additional investigation.

Novel techniques to identify infiltrating tumor cells should also be paired with technologies, such as intraoperative mapping, that allow surgeons to push the boundaries of safe tumor resection in eloquent regions of the brain. Indeed, in a meta-analysis including 8091 patients with a supratentorial glioma, De Witt Hamer et al. showed that patients with their tumors resected using intraoperative mapping had reduced neurological deficits and increased incidence of gross total resection [37]. Intraoperative mapping also continues to improve, with new techniques, including asleep triple motor mapping, in development to enhance the ability for surgeons to carry out maximal safe resections [68].

Outside of surgery, the clear benefit of peritumoral FLAIR resection also raises the question of radiation to these lesions. The 2016 European Organization for Research and Treatment of Cancer (EORTC) guidelines for postoperative radiation treatment following GBM resection describes a target area that is 2 cm from the contrast enhancing lesion, based on GBM recurrence patterns [69]. However this may be inadequate and undertreat a significant number of infiltrating tumor cells depending on the amount, and pattern, of FLAIR abnormality present in a specific patient. There is a paucity of literature evaluating the potential benefit of radiosurgery to the peri-tumoral FLAIR in GBM. Duma et al. reported a study of 174 patients with glioblastoma over 15 years where radiosurgery was used to treat the leading edge of the tumor (defined as the volume of tissue with FLAIR abnormality leading away from the contrast-enhancing tumor or resection cavity) They demonstrated a median survival of 23 months, which is higher than historical controls, suggesting some potential benefit to their treatment protocol and supporting consideration of additional investigation [1,70].

## 5. Conclusions

In summary, peri-tumoral FLAIR signal in patients with GBM contains infiltrating tumor cells that likely contribute to disease recurrence even after the resection of contrast enhancing tumor. Maximal safe resection of both contrast enhancing tumor and non-contrast enhancing FLAIR has been shown to provide a survival benefit in patients with GBM, although variability exists in how peri-lesional non-contrast enhancing tumor is defined in the literature. In addition, the benefit of FLAIR resection should be weighed against the negative prognostic impact of a new postoperative neurologic deficit. Additional investigation in the form of larger multicenter studies with a standardized definition of non-contrast enhancing tumor is required to further define and identify the patient subgroups that most benefit the most from FLAIR resection. Finally, intraoperative brain mapping and novel intraoperative tools for improving tumor resection will play an increasing role in maximal safe resection for patients with GBM.

## Data Availability

Not applicable.
